# Pure mucinous adenocarcinoma of the breast with the rare lymphoplasmacytic infiltration: A case report with review of literature

**DOI:** 10.1002/ccr3.8560

**Published:** 2024-03-26

**Authors:** Yash Hasmukhbhai Prajapati, Vishal Bhabhor, Kahan Samirkumar Mehta, Mithoon Barot, Husen Boriwala, Mohamed Omar

**Affiliations:** ^1^ Department of General Surgery GMERS Medical College and Hospital Vadodara Gujarat India; ^2^ Department of Internal Medicine GMERS Medical College and Hospital Vadodara Gujarat India; ^3^ Faculty of Health science University of Nairobi Nairobi Kenya

**Keywords:** breast cancer, histopathology, lymphoplasmacytic, mastectomy, mucinous carcinoma

## Abstract

**Key Clinical Message:**

A unique ER/PR‐positive mucinous adenocarcinoma of breast but CK7/CK20 negativity. A rare finding of Lymphoplasmacytic Infiltration is noted. Successful treatment included modified radical mastectomy and tamoxifen‐based therapy, leads to remission.

**Abstract:**

Mucinous carcinoma, which is characterized by the presence of abundant extracellular mucin, is a rare and distinctive subtype of invasive breast cancer. This subtype accounts for less than 5% of all invasive breast cancers, with pure mucinous carcinoma representing only around 2%. It is most commonly found in older patients, typically in the sixth to early eighth decade of life. We present the case of a 55‐year‐old female with a 15‐year history of a painless, gradually enlarging palpable lump on her left breast. Upon examination, a 5 × 5 cm lump with well‐defined margins and a hard consistency was found in the lower outer quadrant of the left breast. Histopathological examination confirmed a diagnosis of Mucinous Adenocarcinoma of the Left Breast. The patient underwent modified radical mastectomy and received adjuvant endocrine therapy with tamoxifen due to hormone receptor‐positive status. Mucinous carcinoma of the breast is a unique entity with specific histological features including the presence of extracellular mucin pools. Immunohistochemical staining is crucial for determining hormone receptor status, which can guide treatment decisions. Although surgical intervention is the primary approach, the extent of surgery may vary, ranging from lumpectomy to mastectomy. Adjuvant therapies such as chemotherapy and radiotherapy may also be considered based on individual cases. This case underscores the importance of a multidisciplinary approach in managing rare subtypes of breast cancer, such as mucinous carcinoma. Accurate diagnosis, appropriate surgical intervention, adjuvant therapy, and long‐term follow‐up are critical components of treatment.

## Graphical abstract

Shows the progression of the patient pre operative to after the procedure (Lumpectomy) was performed.

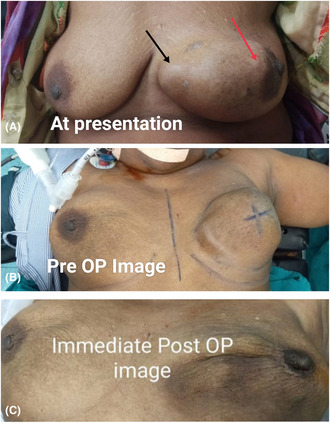

## 1 INTRODUCTION

Mucinous carcinoma represents a distinct subtype of invasive breast cancer. Previously referred to as colloid carcinoma, this unique variant has been characterized by a notable incidence. The majority of studies indicate that less than 5% of all invasive breast cancers possess a mucinous component, with only approximately 2% being pure mucinous carcinoma.^1^, ^2^, ^3^, ^4^ These cases often occur in older patients, typically in the sixth to early eighth decade of life. Although many patients present with palpable tumors, a significant proportion exhibit non‐palpable mammographic abnormalities. These abnormalities manifest as poorly defined or lobulated mass lesions that rarely display calcification.^5^, ^6^, ^7^, ^8^ Notably, approximately 20% of mucinous carcinomas remain occult on mammography.^9^

Mucinous carcinomas exhibit a distinctive appearance upon gross examination. They present as well‐circumscribed tumors with soft, gelatinous consistency, and a glistening cut surface. Occasionally, cases with higher fibrous stroma content may exhibit a firmer consistency. The literature reports a wide range of tumor sizes, with an average of approximately 3 cm.

A recent histological variant of mucinous carcinoma has emerged, characterized by intermediate‐to‐high‐grade nuclei, a hobnail pattern, and micropapillary architecture. This variant, referred to as pure mucinous carcinoma with a micropapillary pattern or mucinous micropapillary carcinoma, has been associated with HER2 positivity and aggressive behavior.^10^, ^11^, ^12^, ^13^

## 2 CASE PRESENTATION

We report this case in line with the updated consensus‐based Surgical Case Report (SCARE) 2020 criteria.^14^

**FIGURE 1:**
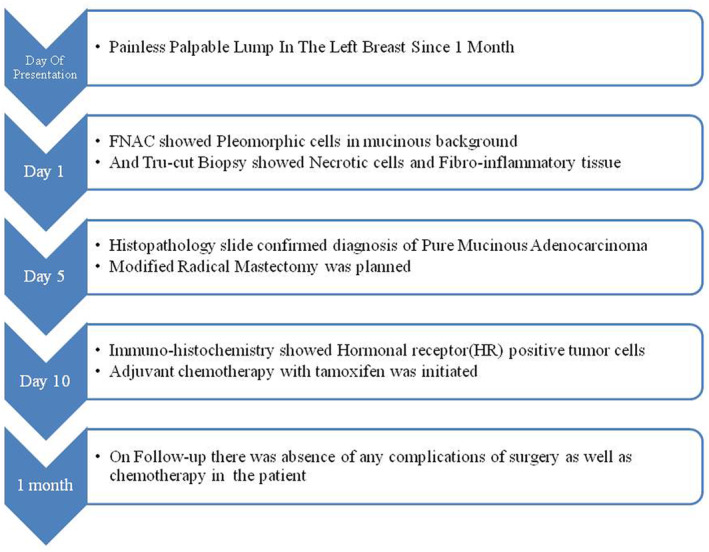
Whole case timeline from presentation to follow‐up.

The complete Case Timeline. (Figure 1).

A 55‐year‐old female presented to the surgical outpatient department with a complaint of a painless palpable lump in her left breast that had persisted for the past month. The lump gradually increased in size over the 15 years. She denied a history of vomiting or bone pain.

**FIGURE 2:**
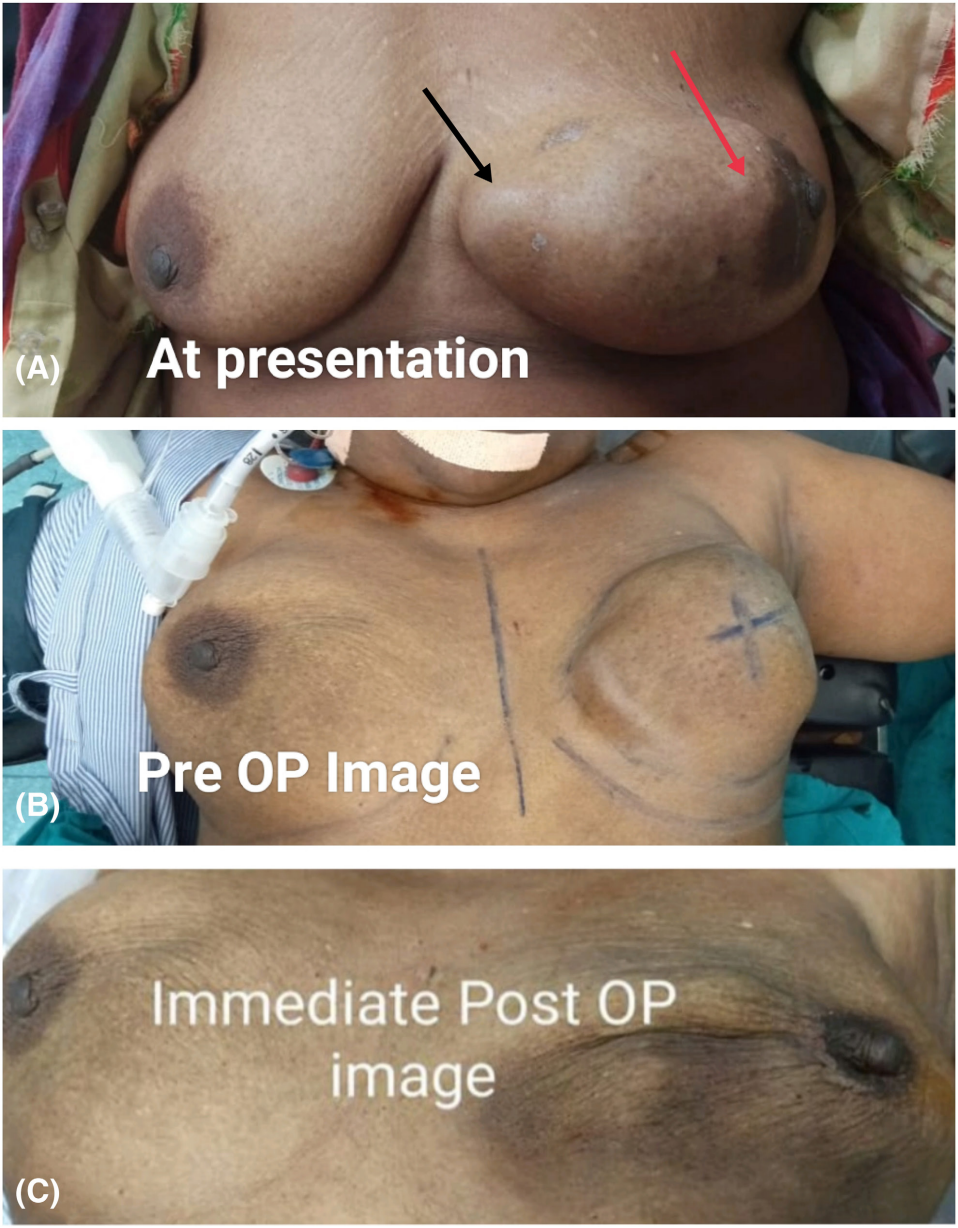
(A) On presentation an enlargement seen on left breast, nodules seen (Black arrow), and nipple–areolar complex displacement seen (Red arrow); (B) Image of preoperative (PRE‐OP) showing cut margins; (C) Immediate postoperative image after lumpectomy.

Upon examination, a lump measuring 5 × 5 cm was found in the lower outer quadrant of the left breast. The lump had uneven dimensions and well‐defined margins (Figure 2). It displayed a hard consistency, a smooth surface, and slight tenderness. The lump did not fluctuate or transilluminate. It appeared to be fixed to the skin and underlying breast tissue. There was no evidence of dimpling or nipple retraction. Palpable axillary lymph nodes were absent and no skin changes were observed. The nipple–areola complex is displaced, with no nipple discharge.

## 3 METHODS (IMAGING AND DIAGNOSTIC WORKUP AND TREATMENT APPROACH)

Given the patient's presentation, a further diagnostic workup was initiated. Mammography was performed to assess non‐palpable mammographic abnormalities associated with the lump. The mammogram revealed poorly defined, lobulated mass lesions with no evidence of calcification, consistent with the features often observed in mucinous carcinomas.

Subsequently, core needle biopsy was performed to obtain tissue for histopathological examination. Fine needle aspiration cytology (FNAC) of the hard left breast lump appeared in the inner quadrant, with a lump measuring 10 × 8 cm. Two milliliters of cystic fluid were focally aspirated after three rounds of FNAC. Hypercellular FNAC smears showed mildly pleomorphic tumor cells in clusters with a mucinous background. Via Tru‐cut biopsy, multiple tissue pieces of 1.0 × 0.3 × 0.2 cm were received and sectioned which shows necrotic and fibro‐inflammatory tissue. An excisional biopsy was performed on the left breast lump and multiple yellowish‐gray firm to soft tissue masses measuring 15 × 7 × 4.5 cm in size. Myxoid mucinous areas are also observed. On cutting open, whitish, brownish‐solid areas with cystic areas. Tumor cells were observed in small clusters, solid nests, and cribriform structures within the mucin pools. The tumor cells showed a high NC ratio, moderate pleomorphism, hyperchromasia, and a scanty cytoplasm. The clinical stage of the tumor was overall grade 1 with AJCC staging of T2N1aMx. Histopathological examination confirmed a diagnosis of mucinous adenocarcinoma of the left breast.

Given the patient's age and tumor characteristics, a multidisciplinary treatment plan was formulated. The patient was deemed suitable for surgical intervention. Lumpectomy was performed presurgically to determine the clinical stage of the tumor as well as trying to excise the tumor while preserving breast tissue. But later on staging, it was found to be of T3N1aMx. Hence, the surgical approach aimed to achieve clear margins and minimize the impact on the aesthetic appearance of the breast. Therefore, a modified radical mastectomy of the left breast was performed using an elliptical Stewart incision and nipple–areola complex, pectoral fascia. Axillary lymph nodes of levels one, two, and three and breast tissue were removed, excluding the pectoralis minor muscle, and only retracted, upon which the specimen was acquired. The gross specimen of the left breast lump showed multiple yellowish‐gray soft tissue masses with firm consistency, measuring 10 × 7 × 4 cm. Myxoid mucinous areas are also observed. On cutting open, whitish, brownish‐solid areas with cystic areas. A left‐sided modified radical mastectomy (MRM) measuring 23 × 19 × 3.5 cm. The overlying skin flap measures 12.5 × 5 cm.

**FIGURE 3:**
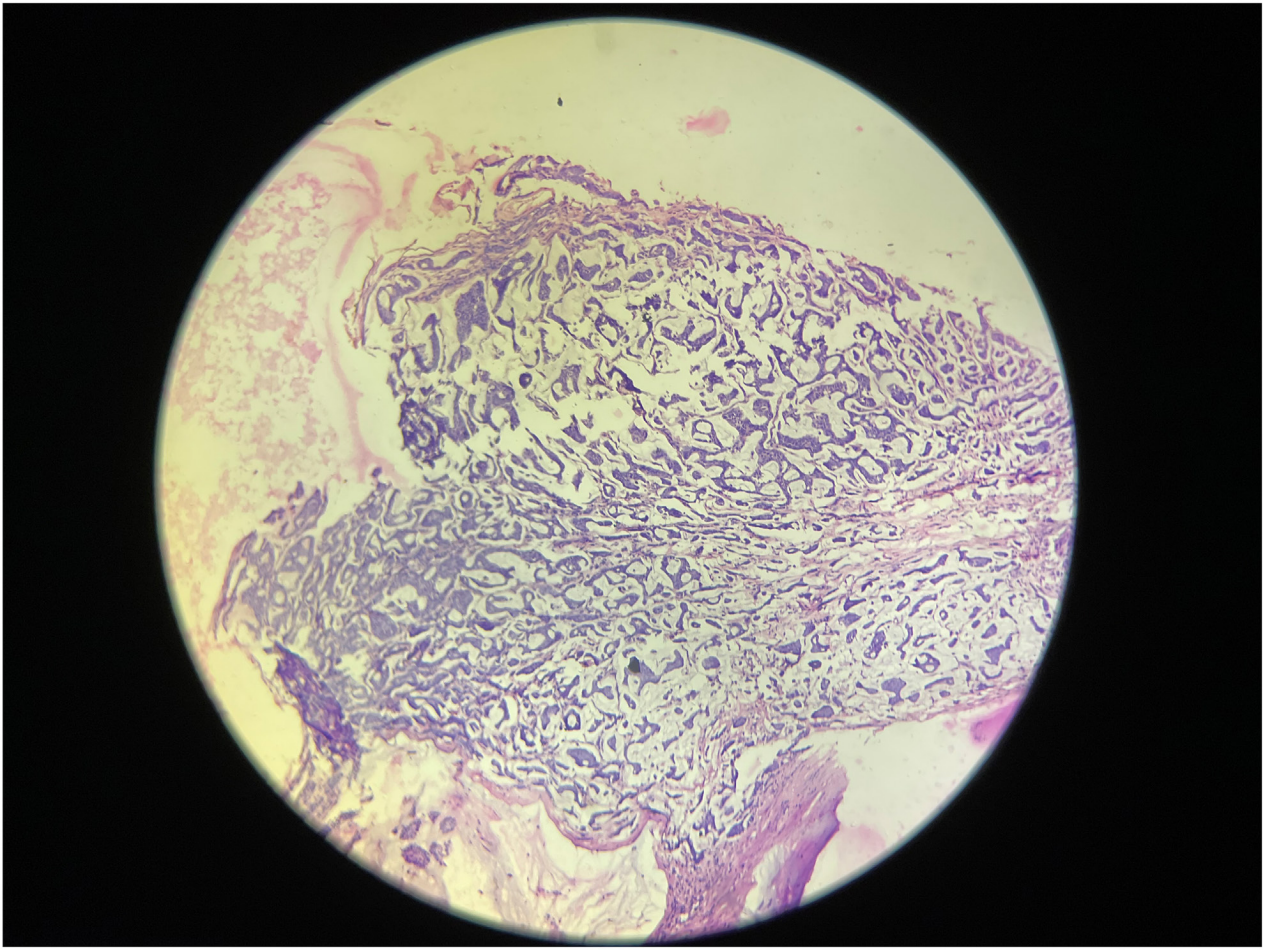
Histopathological section shows clusters of tumor cells “floating in pools of mucin” (H&E stain, ×40).

**FIGURE 4:**
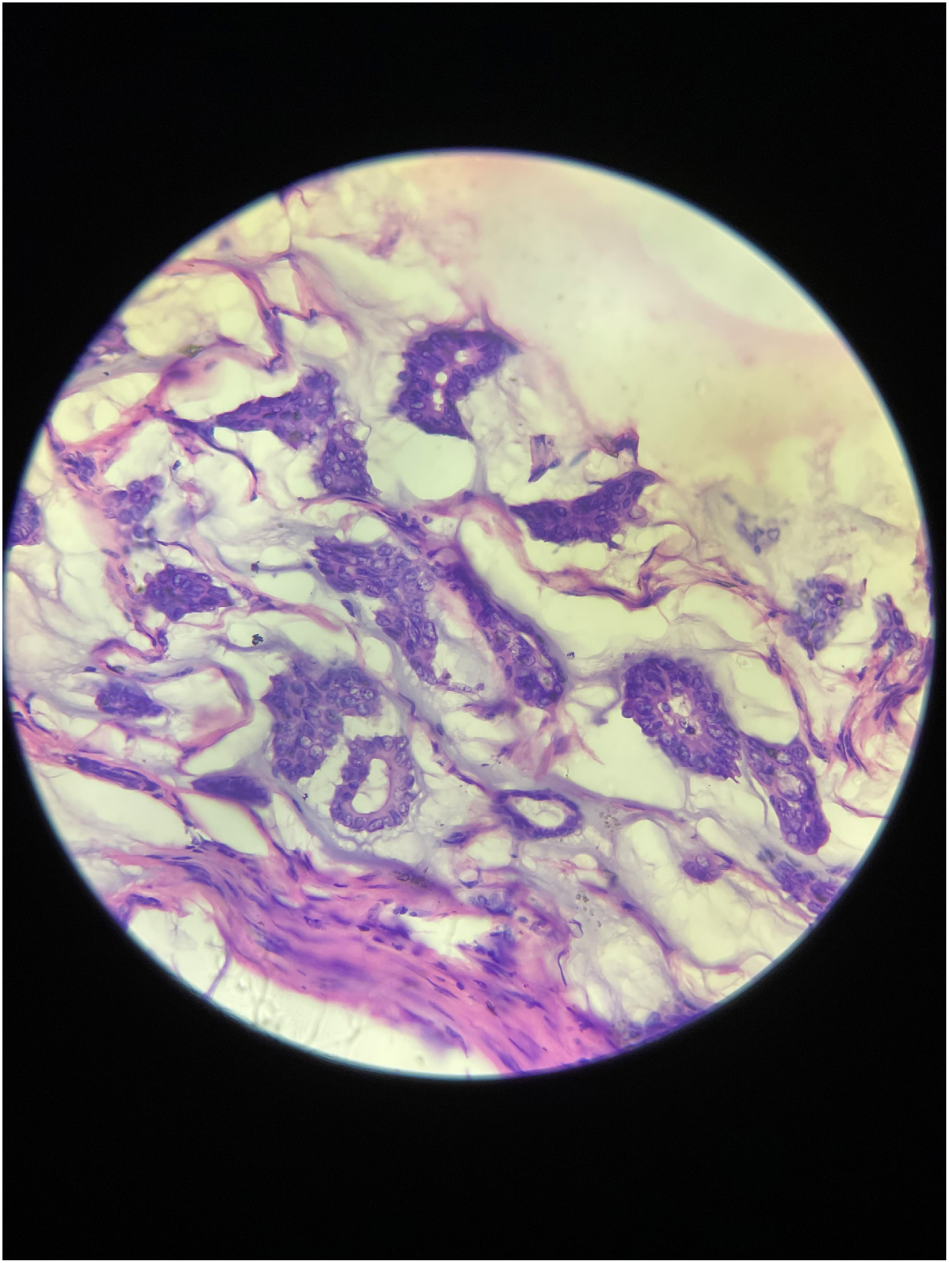
Histopathological section shows tumor cell clusters arranged in acinar and micropapillary patterns. The tumor cells have low‐to‐intermediate‐grade nuclei (H&E stain, ×400).

**FIGURE 5:**
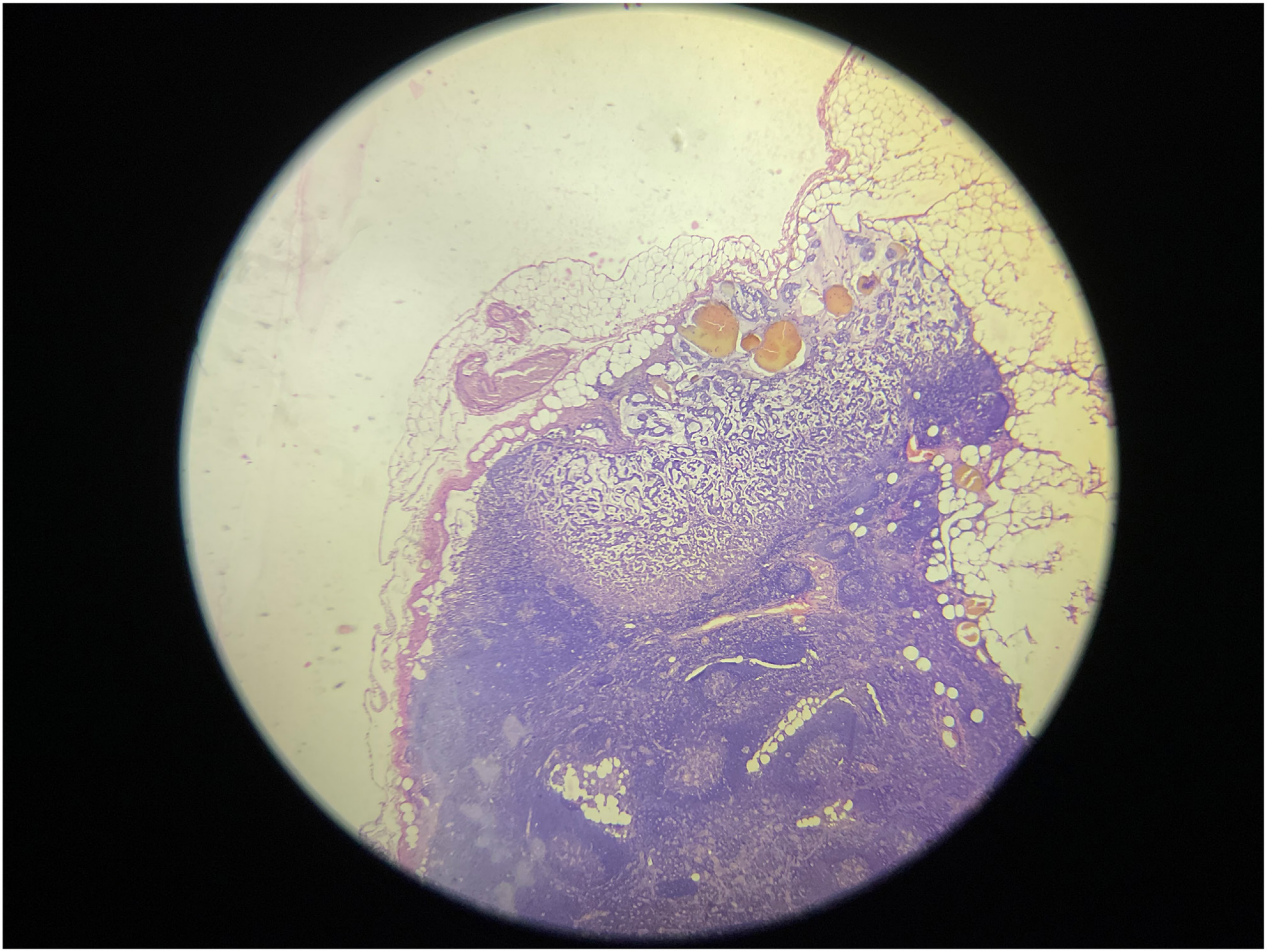
Histopathological section shows mucinous carcinoma metastasis to axillary lymph node (H&E stain, ×40).

Microscopic analysis of the biopsy specimen confirmed the presence of extracellular mucin pools surrounded by capillaries containing fibrous septae. Some tumor cells were also found scattered singly in the mucinous stroma, and mucin pools were separated by fibrous stroma (Figure 3), and at a magnification of 400× (Figure 4). Mild chronic inflammatory infiltrates were also observed. Eleven regional lymph nodes were identified, three of which showed invasion by tumor cells. Lymphovascular and dermal lymphovascular invasion were not present, but lymphoplasmacytic infiltration was identified (Figure 5).

Given the distinct histological subtypes, additional immunohistochemical staining was performed to determine the hormone receptor status of the tumor cells. The results indicated a hormone receptor‐positive status, with tumor cells exhibiting immunoreactivity for both estrogen receptors (ER) and progesterone receptors (PR). Hormone receptor‐positive status indicates a potential benefit from hormone therapy. Therefore, adjuvant endocrine therapy with tamoxifen was initiated as a part of the treatment plan to reduce the risk of recurrence.

### 3.1 Conclusion and results (outcome and follow‐up)

Following successful modified radical mastectomy and initiation of adjuvant therapy, the patient was closely monitored postoperatively for 1 year. Regular clinical examinations, imaging studies, and laboratory tests were scheduled to track progress and detect any signs of recurrence or complications. The patient received comprehensive postoperative care instructions, including wound care and guidelines for managing any potential side effects of adjuvant therapy. She was encouraged to report any new symptoms, discomfort, or concerns to the medical team. Regular follow‐up appointments with her oncologists allowed for the continuous monitoring of her health and well‐being.

## 4 DISCUSSION

Mucinous carcinoma, a distinctive subtype of invasive breast cancer, presents with unique histological and clinical features. The case of a 55‐year‐old female highlights the importance of recognizing the characteristic presentation of mucinous carcinoma, including its clinical, imaging, and histopathological features. Patient management involves a comprehensive approach that encompasses surgical intervention and adjuvant therapy to optimize the outcome and minimize the risk of recurrence. As the field of oncology continues to advance, personalized treatment strategies tailored to the specific characteristics of the tumor and patient's health status remain pivotal in achieving favorable outcomes.

Microscopically, mucinous carcinomas are characterized by the presence of abundant extracellular mucin separated by fibrous septae containing capillaries. Tumor cells are arranged in small clusters, solid nests, or cribriform structures within the extracellular mucin pools. Capella et al.^15^ classified mucinous carcinomas based on their cellularity and identified two types. Paucicellular tumors exhibit ribbon, annular, and cribriform growth patterns (Type A), whereas highly cellular tumors display clumped and sheet‐like growth patterns with less extracellular mucin (Type B). Type B mucinous carcinomas typically exhibit endocrine differentiation, as evidenced by immunoreactivity for chromogranin, synaptophysin, or cytoplasmic argyrophilic granules.

At present, approximately 30 cases of primary mucinous adenocarcinoma have been noted (Table 1) since 1998, and the clinicopathologic features are summarized in the following Table. Their ages ranged from 49 to 96 years of age. Most of them are postmenopausal females. In the table below of the previous cases, 20.83% of cases were found to have ductal carcinoma in situ (DCIS)‐positive tumors, indicating a localized pre‐invasive component in these breast cancer cases. Approximately 13 cases were ductal carcinoma in situ (DCIS)‐positive, six cases were mucinous adenocarcinoma in situ‐positive, and eight cases showed mucus spillover. Our patient was DCIS‐negative, mucinous adenocarcinoma in situ‐positive, and did not show mucus spillover. Lymph node involvement was relatively infrequent, with 12.5% of cases showing lymph node metastasis, suggesting that this subtype of mucinous adenocarcinoma of the breast tends to have a lower propensity for lymphatic spread. Four cases show lymph node mets in prior research. Hormone receptor positivity, as indicated by either estrogen receptor (ER) or progesterone receptor (PR) positivity, was a prevalent feature, with 87.5% of cases exhibiting hormone receptor positivity. Furthermore, all cases in the dataset were reported as HER2‐negative, signifying a lack of HER2 gene amplification; in our case, ER and PR positivity in most cases HER2 gene amplification is rare, and in this case, also HER2Neu receptor with a very low proliferative index.

**TABLE 1:** Clinicopathological analysis of cases in the literature of breast primary mucinous cystadenocarcinoma.

Author's name	Case	Age (years)	Size (mm)	DCIS	MCA in situ	Mucus spillover	Lymphatic Mets	ER	PR	HER2	CK7	CK20	Follow‐up
Lin DL et al.^16^	1	62	5.6	−	−	−	−	−	−	−	+	−	ANED, 5 months
Petersson F et al.^17^	2	73	45	+	−	+	−	−	−	+	+	−	NA
Rosen PP et al.^18^	3	79	60	+	+	+	−	−	−	−	+	−	ANED, 22 months
Honma et al.^19^	4	96	20	−	−	−	+	−	−	−	+	−	DOD, 46 months
Nayak A et al.^20^	5	68	62	+	−	+	−	−	−	−	+	−	ANED, 3 months
Kucukzeybek BB et al.^21^	6	55	20	+	−	−	−	−	−	+	+	−	ANED, 10 months
Koenig C et al.^22^	7	54	190	−	−	−	+	−	−	−	+	−	ANED, 24 months
Chen et al.^23^	8	65	30	+	+	−	−	−	−	−	+	+	ANED, 8 months
Seong M et al.^24^	9	59	20	−	−	−	−	−	−	+	NA	NA	NA
Sentani K et al.^25^	10	65	30	+	+	+	+	−	−		+	−	ANED, 6 months
Koenig C et al.^22^	11	67	23	+	−	+	−	−	−	−	+	−	ANED, 22 months
Wang X et al.^26^	12	66	25	+	+	+	−	−	−	−	+	−	ANED, 10 months
Coyne JD et al.^27^	13	51	40	−	−	−	−	−	−	NA	+	−	NA
Seong M et al.^24^	14	50	22	−	−	−	−	−	−	−	NA	NA	NA
Deng Y et al.^28^	15	41	70	+	+	−	−	−	−	−	+	−	ANED, 24 months
Koenig C et al.^22^	16	49	85	+	+	−	−	−	−	−	+	−	ANED, 11 months
Rakici S et al.^29^	17	52	100	−	−	+	−	+	−	−	−	−	ANED, 24 months
Koenig C et al.^22^	18	61	8	−	−	−	−	−	−	−	+	−	NA
Li XY et al.^30^	19	52	65	−	−	−	−	−	−	−	+	−	ANED, 12 months
Koufopoulos N et al.^31^	20	63	16	−	−	−	+	−	−	−	+	−	ANED, 48 months
Lee SH et al.^32^	21	55	25	+	−	−	−	−	−	−	+	−	ANED, 6 months
Domoto H et al.^33^	22	74	100	+	−	+	−	−	−	−	+	−	ANED, 24 months
Kim SE et al.^34^	23	59	9	+	−	−	−	−	−	−	+	−	ANED, 3 months
Gulwani H et al.^35^	24	61	30	−	−	−	−	−	−	−	+	−	ANED, 6 Months
Current Case	25	55	230	−	+	−	−	+	+	−	−	−	ANED, 3 months

Abbreviations: ANED, alive with no evidence of disease; DCIS, ductal carcinoma in situ; DOD, died of other diseases; MCA in situ, mucinous cystadenocarcinoma in situ; NA, not available.

Most previously reported cases underwent partial or radical mastectomy; in some cases, lumpectomy or mastectomy was performed, while others underwent modified radical mastectomy and received chemotherapy and radiotherapy. However, follow‐up results indicated that the prognosis of patients with MCA is good.

Primary mucinous adenocarcinoma of the breast is rare and histologically similar to primary mucinous adenocarcinomas of the pancreas and ovary. Confirmation of the diagnosis of mucinous adenocarcinoma of the breast requires exclusion of metastasis from the pancreas and ovary. It was excluded by combining clinical manifestations, thorough medical history, and imaging examinations. Immunohistochemical detection of CK7 and CK20 also helps in diagnosis. Additionally, approximately 29.17% of the cases were negative for both CK7 and CK20. Most primary mucinous adenocarcinomas of the pancreas and ovary are positive for CK7 and CK20, and gastrointestinal tumors are CK7‐negative but CK20‐positive. In our case, the primary mucinous adenocarcinoma of the breast was CK7‐ and CK20‐negative, which confirmed the diagnosis and ruled out other possible differentials and the mucinous adenocarcinoma in situ seen in this case, which also supports that the tumor is primarily to the mammary gland.

Lymphoplasmacytic infiltration is related to or consists of lymphocyte and plasma cell infiltration into the tissue. The evaluation of breast lymphoplasmacytic infiltration is problematic. In our case, lymphoplasmacytic infiltration is observed, with negative lymphovascular invasion. Dense infiltration of small B cells and plasma cells often prompts consideration for the mucosa‐associated lymphoid tissue (MALT) type, which is an extranodal marginal zone lymphoma and comprises 23%–64% of all lymphomas of the breast. However, several benign mass‐forming lesions with lymphoplasmacytic infiltrates have been described in the breast as well as a site of involvement for IgG4‐related mastitis, in which sclerosis and dense lymphoplasmacytic infiltrate may efface the underlying tissue and stimulate malignancy. This explains the lymphoplasmacytic involvement observed in this case.

The case of a 55‐year‐old female with mucinous carcinoma underscores the importance of a multidisciplinary approach to the management of breast cancer. Comprehensive evaluation, accurate diagnosis, appropriate surgical intervention, adjuvant therapy, and long‐term follow‐up collectively contribute to optimal patient outcomes and quality of life.

Advances in medical knowledge and technology continue to shape the landscape of breast cancer treatment, enabling tailored approaches that consider individual patient characteristics and tumor biology. As research progresses, further insights into the behavior of mucinous carcinoma and its subtypes may refine treatment strategies and contribute to improved prognosis in patients diagnosed with this unique breast cancer subtype.

Ultimately, the collaborative efforts of healthcare professionals, the dedication of patients to their treatment plans, and ongoing research efforts play a pivotal role in advancing the understanding and management of mucinous carcinoma and enhancing the overall well‐being of those affected by this condition.

## AUTHOR CONTRIBUTIONS

**Yash Hasmukhbhai Prajapati:** Conceptualization; methodology; writing – original draft; writing – review and editing. **Vishal Bhabhor:** Resources; supervision; validation; writing – review and editing. **Kahan Samirkumar Mehta:** Conceptualization; funding acquisition; writing – original draft; writing – review and editing. **Mithoon Barot:** Resources; software; supervision. **Husen Boriwala:** Writing – original draft; writing – review and editing. **Mohamed Omar:** Resources; supervision; validation.

## FUNDING INFORMATION

No funding sources.

## CONFLICT OF INTEREST STATEMENT

None declared.

## CONSENT

Written informed consent was obtained from the patient to publish this report in accordance with the journal's patient consent policy.

## Data Availability

The data that support the findings of this study are available from the corresponding author upon reasonable request.
